# Calmodulin variants associated with congenital arrhythmia impair selectivity for ryanodine receptors

**DOI:** 10.3389/fmolb.2022.1100992

**Published:** 2023-01-05

**Authors:** Giuditta Dal Cortivo, Valerio Marino, Silvia Bianconi, Daniele Dell'Orco

**Affiliations:** Department of Neurosciences, Biomedicine and Movement Sciences, Section of Biological Chemistry, University of Verona, Verona, Italy

**Keywords:** point mutation, kinetics, kinetic discrimination, molecular dynamics, protein structure network, protein-protein recognition, free-energy

## Abstract

Among its many molecular targets, the ubiquitous calcium sensor protein calmodulin (CaM) recognizes and regulates the activity of ryanodine receptors type 1 (RyR1) and 2 (RyR2), mainly expressed in skeletal and cardiac muscle, respectively. Such regulation is essential to achieve controlled contraction of muscle cells. To unravel the molecular mechanisms underlying the target recognition process, we conducted a comprehensive biophysical investigation of the interaction between two calmodulin variants associated with congenital arrhythmia, namely N97I and Q135P, and a highly conserved calmodulin-binding region in RyR1 and RyR2. The structural, thermodynamic, and kinetic properties of protein-peptide interactions were assessed together with an in-depth structural and topological investigation based on molecular dynamics simulations. This integrated approach allowed us to identify amino acids that are crucial in mediating allosteric processes, which enable high selectivity in molecular target recognition. Our results suggest that the ability of calmodulin to discriminate between RyR1 an RyR2 targets depends on kinetic discrimination and robust allosteric communication between Ca^2+^-binding sites (EF1-EF3 and EF3-EF4 pairs), which is perturbed in both N97I and Q135P arrhythmia-associated variants.

## 1 Introduction

**FIGURE 1:**
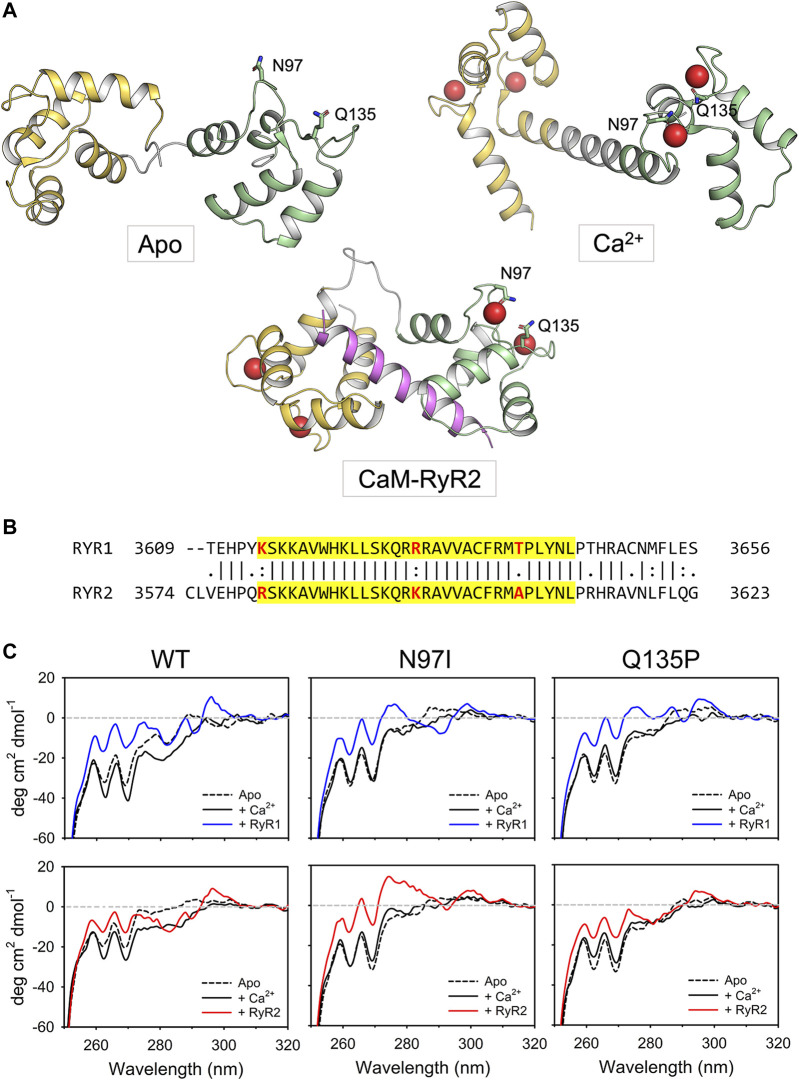
Tertiary and quaternary structure of CaM variants in complex with RyR1/2 peptides. **(A)** Cartoon representation of CaM in three different conformational states, namely apo (PDB: 1DMO (Zhang et al., 1995)), Ca^2+^-bound (PDB: 1CLL (Chattopadhyaya et al., 1992)) and complexed with the RyR2 peptide used in this study (PDB: 6Y4O (Holt et al., 2020)). The N-terminal domain is colored in yellow, the linker region in grey, while the C-terminal domain in green. The RyR2 peptide is represented in magenta. Ca^2+^ ions are represented as red spheres while the side chains of N97 and Q135 are represented in sticks with C atoms colored according to the structural region, O atoms in red and N atoms in blue. **(B)** Pairwise sequence alignment of the Calmodulin Binding Domain-2 of human RyR1 (Uniprot entry P21817) and RyR2 (Uniprot entry Q92736). The sequence relative to the two RyR peptides employed in this study is highlighted in yellow, the residues that are not identical in such region are represented in bold and colored in red. **(C)** Near-UV CD spectra (250–320 nm) of 50 μM CaM were collected in the presence of 500 µM EGTA (black dashed line) and after sequential additions of 1 mM Ca^2+^ (black solid line, 500 µM free Ca^2+^) and 100 μM RyR peptides (blue solid line for RyR1, red solid line for RyR2). The spectrum of sole buffer was considered as blank and subtracted; each curve represents the average of five accumulations. Temperature was set at 25°C and signal was normalized to protein concentration.

Calmodulin is a widely expressed calcium sensor protein capable of binding up to four Ca^2+^ ions with micromolar affinity, thereby acquiring conformations that allow specific recognition of more than 300 molecular targets (Stevens, 1983). The two globular domains of CaM are individually composed by a pair of helix-loop-helix EF-hand motifs, each of which has a different affinity for Ca^2+^. The N-terminal domain, consisting of the EF1 and EF2 motifs, shows lower affinity for Ca^2+^ (K_D_ ∼10 μM) than the C-terminal domain (K_D_ ∼1 μM), consisting of the EF3 and EF4 motifs (Figure 1A). Ca^2+^ ion binding occurs with positive cooperativity within each domain, but in the absence of a molecular target, no interdomain cooperativity is observed (Linse et al., 1991). Indeed, binding of a target to CaM induces substantial changes in the apparent affinity and cooperativity for Ca^2+^ binding (Newman et al., 2008; Theoharis et al., 2008; Valeyev et al., 2008), which is closely related to the high structural plasticity of CaM (Astegno et al., 2014). Recognition of a wide variety of molecular targets gives CaM the ability to regulate diverse biochemical processes, such as transmembrane ion transport, cell mobility and proliferation, apoptosis, cytoskeleton remodeling, and protein folding (Chin and Means, 2000; Carafoli, 2002). This versatility is the result of both CaM’s very high sensitivity in detecting even minute changes in intracellular Ca^2+^ concentration and remarkable selectivity in target activation.

Ryanodine receptors (RyR), sentinels of massive intracellular Ca^2+^ stores contained in the sarcoplasmic reticulum, release Ca^2+^ into the cytosol in response to sarcolemmal depolarization, thereby facilitating mobilization of the myofilaments and enabling cell contraction in cardiac and skeletal muscle cells. To achieve cell relaxation, Ca^2+^ must be rapidly resequestered or extruded from the cytosol (Capes et al., 2011). CaM is one of the proteins that precisely regulate the activity of RyR1 and RyR2 receptors, which are predominantly expressed in skeletal and cardiac muscle, respectively (Capes et al., 2011). Because of its trace-level expression in the heart, the contribution of RyR1 to cardiac function is currently unknown, while that of RyR2 is much better understood. In fact, although RyR2 is also strongly expressed in neurons and in visceral and arterial smooth muscle, it is in cardiomyocytes that the essential action of CaM in channel regulation has been accurately established. Even though RyR2 can bind Ca^2+^, it is precisely the interaction with CaM that allows fine-tuning of channel inhibition, which is strictly dependent on the concentration of free Ca^2+^ (Sorensen et al., 2013; Van Petegem, 2015). This enables the proper dynamics of Ca^2+^-induced Ca^2+^ release (CICR) mechanism, a key step in the regulation of the excitation-contraction process in cardiomyocytes (Sorensen et al., 2013).

Dysregulation of Ca^2+^ release *via* RyRs is associated with life-threatening diseases in both skeletal and cardiac muscle (Capes et al., 2011). This became particularly evident 10 years ago, when two missense mutations in CaM were found to be associated with heart failure and sudden cardiac death due to Catecholaminergic Polymorphic Ventricular Tachycardia (CPVT) and long QT syndrome (LQTS) (Nyegaard et al., 2012). To date, the number of point mutations in any of the three genes (*CALM1-3*) encoding CaM in humans and associated with heart diseases has risen to 17 (Jensen et al., 2018). On the other hand, structural knowledge has been accumulating on the CaM-RyR2 interaction (Sondergaard et al., 2017; Brohus et al., 2019; Chi et al., 2019; Sondergaard et al., 2019; Holt et al., 2020; Sondergaard et al., 2020). Unprecedented insight into the functional properties of RyR2 and its regulation by CaM has come from cryogenic-electron microscopy (cryo-EM) (Peng et al., 2016; Chi et al., 2019; Gong et al., 2019), which shed light on the three previously identified CaM-binding domains (CaMBD) shared by RyR receptors, which could interact individually or in groups with CaM lobes to regulate channel function (Lau et al., 2014). Cryo-EM revealed a complex CaM-RyR2 recognition mechanism, in which apo- and Ca^2+^-bound CaM bind to distinct but overlapping RyR2 sites, and demonstrated that Ca^2+^-bound CaM is one of many possible regulators competing for RyR2 gating (Gong et al., 2019). Among the possible CaM-RyR binding interfaces, a common region was identified by structural investigations, which corresponds to the K3614-L3644 stretch in RyR1 and the R3581-L3611 stretch in RyR2 (Lau et al., 2014). This finding raises the question of why an interaction based on an extremely similar region of the two targets (3 different amino acids out of 31; Figure 1B) results in a severe and life-threatening phenotype only in the case of the interaction with RyR2, which is specific to cardiac muscle cells. In a more general context, it is important to identify the molecular determinants that allow CaM to discriminate among different targets, even when selective recognition is based on a few different amino acids.

To unravel the molecular mechanisms underlying the selective perturbation of the recognition between CaM and RyR1 or RyR2 in arrhythmia-associated conditions, we focused on a CaM binding target region comprising 31 amino acids with a very similar sequence in the two RyRs (known as CaMBD2). We conducted a comprehensive biophysical investigation of the structural, kinetic, and thermodynamic properties underlying the association between peptides comprising the analogous RyR1/RyR2 regions and three CaM variants, namely the wild type protein and the N97I and Q135P variants (Figure 1A), which are associated with LQTS and LQTS/CPVT, respectively (Jensen et al., 2018). Integrating spectroscopic investigation with molecular dynamics simulations and protein structure network analysis, we found that despite the very high sequence similarity of the two RyR1/2 interacting regions, these disease-associated CaM mutations alter CaM selectivity for the specific RyR channel.

## 2 Materials and methods

### 2.1 Plasmid and peptide preparation

The gene encoding for human wild type (WT) calmodulin (Uniprot entry: P0DP23) was cloned into a pET24 (+) vector (resistance to kanamycin); cloning, codon optimization, point mutations and sequence check was done by Genscript. WT and disease-related CaM variants, namely N97I and Q135P, were expressed as His-tag proteins, as in (Dal Cortivo et al., 2022). The nomenclature used in this paper is referred to the mature protein that lacks the Met in position 1. RyR1 and RyR2 peptides, encompassing regions K3614-L3644 (KSKKAVWHKLLSKQRRRAVVACFRMTPLYNL) and R3581-L3611 (RSKKAVWHKLLSKQRKRAVVACFRMAPLYNL) of the human RyR1 and RyR2 channels respectively, were purchased from Genscript as lyophilized powder (purity >95%, assessed *via* mass spectrometry and HPLC). From now on, RyR1 and RyR2 refer to the two respective peptides.

### 2.2 Protein expression and purification

The expression and purification of CaM variants were performed as described previously (Dal Cortivo et al., 2022). Briefly, after the transformation of *E. coli* BL21-DE3 strain by thermal shock, cells were grown at 37°C until the OD_600_ reached 0.6. After the expression induction (1 mM IPTG) cells were grown for 4 h at 37°C. Cells were then lysed using lysozyme and DNAse and, after centrifugation, the soluble fraction was loaded onto a His-trap FF crude column (GE Healthcare). After a one-step elution, the His-tagged CaM was dialyzed to allow the cleavage of His-tag at 25°C, overnight, using Tobacco Etch Virus protease (TEV, Promega) in a ratio TEV: CaM of 1U: 450 µg. Reverse immobilized metal affinity chromatography was performed, allowing the collection of the tag-free protein in the flow-through. Purified proteins were then quantified *via* Bradford assay, using a calibration line specific for human CaM (Alphalyze), aliquoted, flash frozen in liquid N_2_ and stored at −80°C until use.

### 2.3 Circular dichroism spectroscopy

Secondary and tertiary structure of CaM variants were assessed using a Jasco-710 spectropolarimeter supplied with a Peltier-type cell-holder as previously detailed (Dal Cortivo et al., 2022). Briefly, far UV (200–250 nm) circular dichroism (CD) spectra were collected both using 10 µM of sole CaM or in co-presence of 20 µM RyR peptides. Spectra of isolated CaM variants and CaM-RyR1/2 complexes were measured in the absence of cations (apo conditions; 300 µM EGTA) and after the addition of saturating Ca^2+^ (600 μM, 300 µM free Ca^2+^) using a 0.1 cm path length quartz cuvette. Near UV CD spectra of 50 µM CaM were collected in the presence of 500 µM EGTA (apo) and after sequential additions of 1 mM Ca^2+^ (500 µM free Ca^2+^) and 100 µM RyRs using 1 cm path length quartz cuvette. Spectra were normalized based on protein concentration measured by Bradford assay and molecular weight of the formed complex. Each spectrum represents the average of five accumulations and the spectrum of the working buffer (20 mM Tris pH 7.5, 150 mM KCl, 1 mM DTT) was considered as reference and subtracted. Temperature and time response were set at 25°C and 4 s, respectively.

### 2.4 Limited proteolysis

Susceptibility of CaM variants to proteolysis was assessed incubating 26.4 µM of protein with 0.4 µM Trypsin (Sigma), i.e. at a protein:trypsin ratio 66:1, in the presence of 2 mM EGTA or Ca^2+^. After 10 min incubation at 25°C (conditions previously optimized to maximize differences in proteolytic patterns (Dal Cortivo et al., 2022)), reactions were blocked adding sample buffer 4x and boiling for 10 min. Each reaction product, together with the untreated sample, was loaded on a 15% SDS-PAGE run at 200 V for 45–50 min and Coomassie blue-stained.

### 2.5 Fluorescence titrations

The apparent affinity of CaM variants for RyR1 and RyR2 was assessed following the intrinsic fluorescence of the only Trp residue present in both peptides. Briefly, 1 µM of each peptide was incubated with increasing concentrations of CaM (0–4 µM) using 20 mM Tris pH 7.5, 150 mM KCl, 1 mM DTT and 100 µM Ca^2+^ as working buffer. The fraction of peptide bound (fb) to CaM (Astegno et al., 2016) as function of the protein concentration, and was calculated as follows:

$$fb=\frac{y-y_{0}}{y_{\max}-y_{0}}$$

where y_0_ and y_max_ are the wavelengths at which the isolated peptide and the saturated complex showed their maximum fluorescence intensity, respectively. Each titration was repeated in triplicate. Each dataset was fitted to a one-site saturation ligand binding function to obtain individual K_D_ values for each replica.

### 2.6 Isothermal titration calorimetry

Isothermal titration calorimetry (ITC) measurements were performed as previously elucidated (Dal Cortivo et al., 2022). Briefly, the MicroCal PEAQ instrument was set to 25°C to perform titrations of RyR1 and RyR2 peptide (125 µM loaded in the syringe) with 10 μM WT-, N97I- and Q135P-CaM in 20 mM Tris pH 7.5, 150 mM KCl, 1 mM DTT, 5 mM Ca^2+^ working buffer. Thirty 1-µL injections were performed, with 150 s delay between each injection and setting the stirring to 750 rpm; each titration was performed at least in triplicate. Dilution effect was considered as blank and subtracted; it was measured by titrating the peptide with sole buffer. Data were fitted to a “one set of sites” model to obtain the number of binding sites (N), the dissociation constant (K_D_) and the enthalpy change (ΔH). These parameters were then used to calculate ΔG, the entropy change (ΔS) and ΔΔG as follows:

$$∆G=RTlnK_{D}=∆H-T∆S$$

$$∆∆G={∆G}_{mut}-{∆G}_{WT}$$

### 2.7 Surface plasmon resonance

Surface plasmon resonance (SPR) experiments were performed using a SensiQ Pioneer instrument and His-Cap sensor chips (from FortèBio and Sartorius). Following a previously optimized protocol (Dal Cortivo et al., 2019; Dal Cortivo et al., 2022) 1000 RU (1 RU = 1 pg mm^−2^) of each His-CaM variant were immobilized on the surface of the sensor chip using 20 mM Tris pH 7.5, 150 mM KCl, 0.005% Tween 20 as working buffer. Immobilized His-CaM variants were washed overnight using a flowrate of 5 μL/min to remove unbound proteins. DTT (100 µM) and Ca^2+^ (5 mM) were freshly added before the beginning of titration experiments. Titrations were performed by injecting increasing concentrations of RyR1 and RyR2 (ranging from 250 nM to 3–4 µM) for 60 s and following the dissociation for 300 s using a flowrate of 20 μL/min. Data were fitted using a 1:1 Langmuir model. The dissociation phase was considered first to obtain *k* ^*off*^ values that were used to calculate the *k* ^*on*^ in the same binding process, according to a pseudo-first-order kinetic scheme. Each titration was repeated at least in triplicate.

### 2.8 Molecular modeling of CaM-RyR peptide complexes

The molecular modeling of Ca^2+^-loaded human CaM complexed to RyR1 and RyR2 peptides was performed with the BioLuminate interface of Maestro chemical simulation suite (v. 12.5.139, Schroedinger) starting from the high-resolution (1.84 Å) X-ray structure of CaM-RyR2 peptide (residues S3582-M3605) complex, with Protein Data Bank entry: 6Y4O (Holt et al., 2020). RyR2 residues K3581, T3606, and P3607 were modelled by BioLuminate to maximize the sequence coverage of the peptide used for *in vitro* experiments. The CaM-RyR2 peptide complex structure was then subjected to the *Protein preparation* pipeline provided by BioLuminate, previously detailed in (Dal Cortivo et al., 2022), which consisted of: i) assignment of bond orders (comprising 0-order bonds to ions) according to the Chemical Components Dictionary database; ii) addition of H atoms; iii) modeling of the missing loop (residues K77-D80) using *Prime*; iv) sampling of the orientation of water molecules; v) calculation of the protonation states of ionizable residues at neutral pH (7.5) using PROPKA; vi) H-bond optimization. Arrhythmia-associated CaM variants N97I and Q135P were introduced by BioLuminate *Mutate residue* tool after selecing the highest-ranked non-clashing rotamer. Analogously, modeling of RyR1 peptide (residues R3614-P3640) was achieved by introducing three mutations (underlined) in RyR2 peptide (residues K3581-P3607), resulting in the following sequence: RSKKAVWHKLLSKQRKRAVVACFRMAP.

### 2.9 Molecular dynamics simulations and *in silico* analysis of CaM-RyR peptide complexes’ stability

CaM-RyR1/2 peptide complexes were subjected to all-atom molecular dynamics (MD) simulations on GROMACS (v. 2020.3) simulation package (Abraham et al., 2015), using CHARMM36 m as forcefield (Huang et al., 2017). Protein complexes were placed in the center of a dodecahedral simulation box containing ∼36,000 atoms, their charge was neutralized with 150 mM KCl, then structures underwent energy minimization and serial 2-ns equilibration in NVT and NPT ensembles, as previously elucidated (Marino et al., 2015). Four 300-ns simulations at 1 atm constant pressure and 310 K constant temperature were performed for each combination of variant and peptide. The convergence and consistency of the conformational space sampled by the simulations was assessed by Principal Component Analysis as described in (Borsatto et al., 2019; Marino and Dell’Orco, 2019). The diagonalization of the covariance matrix calculated on the coordinates of α-carbons (Cα) allows to identify the directions (eigenvectors) of the largest collective motions (eigenvalues) of the simulated systems. Thus, the consistency of the conformational space sampled by each replica was assessed by projecting the frames from the individual 300-ns trajectories onto the first two principal components of the concatenated trajectory. Such projections were classified using Linear Discriminant Analysis, a method that reduces the dimensional space, maximizes intercluster distances, and minimizes intracluster distances. Moreover, the first 20 principal components of each replica and the concatenated trajectories were considered the essential subspace and compared using RMSIP metric, which is defined as follows:

$$RMSIP=\sqrt{\frac{1}{S}\sum_{n=1,\;m=1}^{S}{\left(v_{n}^{i}{∙v}_{m}^{j}\right)}^{2}}$$

where *S* is the number of principal components, $v_{n}^{i}$ and $v_{m}^{j}$ identify the *n* ^th^ and *m* ^th^ principal components of trajectories *i* and *j*.

The Root-Mean Square Fluctuation (RMSF) of Cα and Ca^2+^-ions in each CaM EF-hand (indicative of the structural flexibility of the protein and the apparent affinity for Ca^2+^, respectively), was calculated with respect to the average structure of the complex by means of GROMACS’ *gmx rmsf* function.

The final frames of each of the four 300-ns trajectories of WT CaM-RyR1 and WT CaM-RyR2 complexes were subjected to the *Residue scanning* analysis (provided by BioLuminate) to evaluate the effects of arrhythmia-associated mutations on the Gibbs free energy of protein-peptide folding (ΔΔ $G_{f}^{\mathrm{app}}$ ) and binding (ΔΔ $G_{b}^{\mathrm{app}}$ ), with respect to the WT. This tool computes the mutation-specific thermodynamic cycle based on the Molecular Mechanics/Generalized Born and Surface Area continuum solvation (MM/GBSA). Although this method does not include the contribution of conformational changes, it provides apparent free energy variations (ΔΔG_app_, in kcal/mol) which can reliably estimate mutation-associated differences in stability and affinity, rather than precise thermodynamic measurables.

### 2.10 Protein structure network analysis

All-atom MD simulations provide dynamical structural information, which was condensed in a static Protein Structure Network (PSN) using PyInteraph (Tiberti et al., 2014) with the default parameters for angles and distances cut-off. In detail, H-bonds, electrostatic and hydrophobic interactions were translated into distance and angle constraints between two residues, and the percentage of the trajectory in which such constraints were satisfied was calculated by PyInteraph. Then, if such percentage was higher than the persistence threshold (pT), which is based on the size of the largest hydrophobic cluster, the interaction was translated into an edge between the nodes (residues) of the PSN, otherwise the interaction was considered transient and thus discarded. For further details see Refs (Marino and Dell’Orco, 2016; Marino and Dell’Orco, 2019).

The topology of the PSN was analyzed by means of the degree centrality (number of edges reaching a node) to identify the residues involved in the highest number of interactions (hubs) and thus entitled to preserve protein folding and mediating inter/intramolecular communication of state-specific structural information. Differences in hub degree were calculated with respect to the degree of the same residue in the WT, hubs were defined as nodes with at least six edges in at least one of the tested cases.

Regardless of their structural position, intra/intermolecular signaling between two residues of any binding event or conformational change occurs through persistent non-bonded interactions. Therefore, we monitored the differences in intramolecular communication between EF-hands (identified by their representative bidentate Ca^2+^ coordinating residues E31 (EF1), E67 (EF2), E104 (EF3) and E140 (EF4)) using the previously defined Communication Robustness index (Marino and Dell’Orco, 2016), which is calculated as follows:

$$CR\left(XY\right)=\frac{nXY∙pT}{l}$$

where nXY is the number of shortest paths (of length l) between residues X and Y and pT is the persistence threshold used to filter out transient non-bonded interactions.

## 3 Results

### 3.1 Effects of Ca^2+^ and RyR1/2 peptides on secondary and tertiary structure of CaM variants

Like any other calcium sensor protein, CaM is expected to change its secondary and tertiary structure upon binding to Ca^2+^. Furthermore, interaction of CaM with an essentially unstructured target peptide can induce a specific folding of the latter, as observed in previous studies (Astegno et al., 2016; Dal Cortivo et al., 2022). To assess the structural consequence of Ca^2+^ binding to the three CaM variants in the presence and in the absence of RyR1 and RyR2 peptides we used circular dichroism (CD) spectroscopy.

Near UV CD spectra (Figure 1C) provide information as to the microenvironment of aromatic residues, which are essentially located in the hydrophobic core of a folded protein, and thus represent a fingerprint of protein tertiary structure. CaM lacks tryptophan (W) residues, therefore the apo-to Ca^2+^-bound transition of the isolated protein was characterized by minor spectral changes in the phenylalanine (F) and tyrosine (Y) bands. Addition of Ca^2+^ led to appreciable spectral changes in the Y region for WT and N97I variants, while the change was less pronounced for the Q135P variant (Figure 1C). Interestingly, addition of RyR1 (blue line) or RyR2 peptides (red line) significantly increased the dichroic signal in the F bands, which became less negative for all three variants. A small positive band in the W region was now visible for all three variants, which could be attributed to the burial of the peptide’s W residue upon interaction with CaM. Conversely, the Y region in the presence of RyR1/2 peptides showed a similar pattern in the case of WT CaM, which was significantly perturbed in the mutants’ spectra (Figure 1C). Indeed, a fully positive band was observed for N97I in the presence of RyR2 while interaction with the same peptide led to essentially unperturbed spectrum in the case of Q135P, at odds with the effect observed with RyR1, which significantly affected the Y band in both CaM variants. Since both RyR1 and RyR2 peptides possess one Y residue located far from the structural interface (Holt et al., 2020), this effect seems to be related to a slight conformational change occurring at the level of protein tertiary structure rather than at the level of the protein-peptide assembly.

**FIGURE 2:**
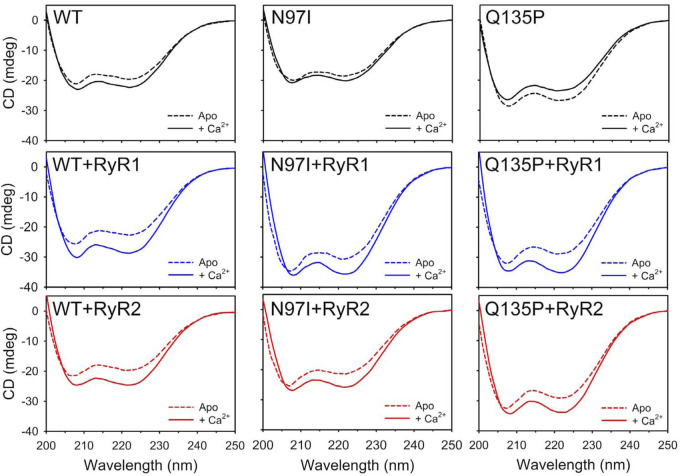
Investigation of secondary structure of CaM variants and their RyR1/2 peptide complexes. Far-UV CD spectra (200–250 nm) of 10 μM CaM variants alone (top panels, black), and incubated with 20 μM RyR1 (center panels, blue), or 20 μM RyR2 (bottom panels, red), were collected in the presence of 300 µM EGTA (dashed lines) and after the addition of 600 µM Ca^2+^ (300 µM free Ca^2+^, solid lines). The spectrum of sole buffer was considered as blank and subtracted; each curve represents the average of five accumulations. Temperature was set at 25°C.

CD spectroscopy in the far UV region was used to monitor changes in protein secondary structure upon Ca^2+^- and peptide-binding (Figure 2), which in many calcium sensor proteins is accompanied by an increase of the dichroic signal attributed to an increased α-helix content or to the achievement of a more compact structure (Astegno et al., 2014; Marino et al., 2015). Spectral shape was found to depend on the CaM variant (Supplementary Table S1). As observed in our recent study (Dal Cortivo et al., 2022) the two minima at 208 and 222 nm typical of a mainly α-class protein shifted to more negative values upon addition of Ca^2+^ for WT and N97I CaM (Δθ_222_/*θ*_222_ = 18% and 8.6%, respectively) at odds with Q135P which, similarly to other variants carrying point mutations in the EF4 motif, decreased the intensity of the spectrum upon Ca^2+^-binding (Δθ_222_/*θ*_222_ = −11.6%), suggesting a loss of α-helix content and/or a less compact structure (Figure 2). However, addition of either RyR1 or RyR2 peptides dampened such differences, as all three variants displayed an increase of the dichroism signal (Figure 2, Supplementary Table S1) and a transition towards a coiled-coil conformation as indicated by the *θ*_222_/*θ*_208_ values approaching unity in the presence of Ca^2+^ (*θ*_222_/*θ*_208_ ranging from 0.96 to 1.02, Supplementary Table S1) as well as a more similar Δθ_222_/*θ*_222_ value (ranging from 16.7% to 22.3%), indicative of the binding-induced folding of the peptide into an α-helix secondary structure.

**FIGURE 3:**
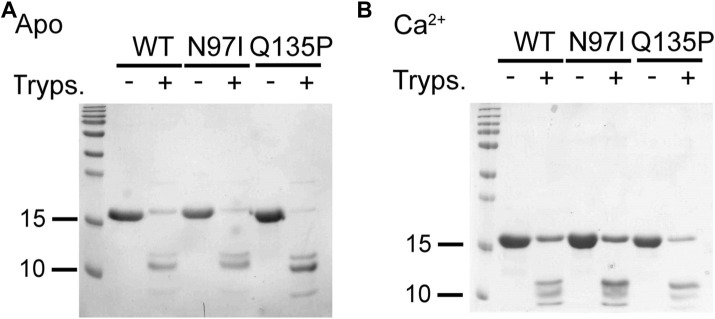
Limited proteolysis of CaM variants. CaM variants (26.6 µM) were incubated with .4 µM trypsin (ratio 1:66 trypsin: CaM) in the presence of 2 mM EGTA **(A)** or Ca^2+^ **(B)**. Reactions were run for 10 min at 25°C and then blocked by boiling samples for 10 min at 96°C. Proteolytic fragments were loaded on a 15% SDS-PAGE and Coomassie blue-stained. For each investigated condition, the untreated protein was loaded as a control.

Digestion with trypsin of isolated CaM variants (Figure 3) confirmed the stabilizing role of Ca^2+^ binding, as judged by the presence of undigested bands for all three variants, which was more pronounced in the presence of Ca^2+^ than in the apo form (Figure 3A,B). Moreover, proteolytic patterns suggested that Q135P and N97I CaM are less stable than the WT in the absence of Ca^2+^ as shown by the higher intensity of the bands at low molecular mass. Moreover, Q135P appeared to be the less stable variant, regardless of the presence of Ca^2+^.

Overall, these data suggest that the N97I and Q135P substitutions induced small but detectable conformational changes in the secondary and tertiary structure of CaM, and the binding of either RyR1 or RyR2 peptide occurred in any case, leading to similar quaternary structures.

### 3.2 Affinity for RyR1 and RyR2 peptides of CaM variants assessed by fluorescence spectroscopy and isothermal titration calorimetry

**FIGURE 4:**
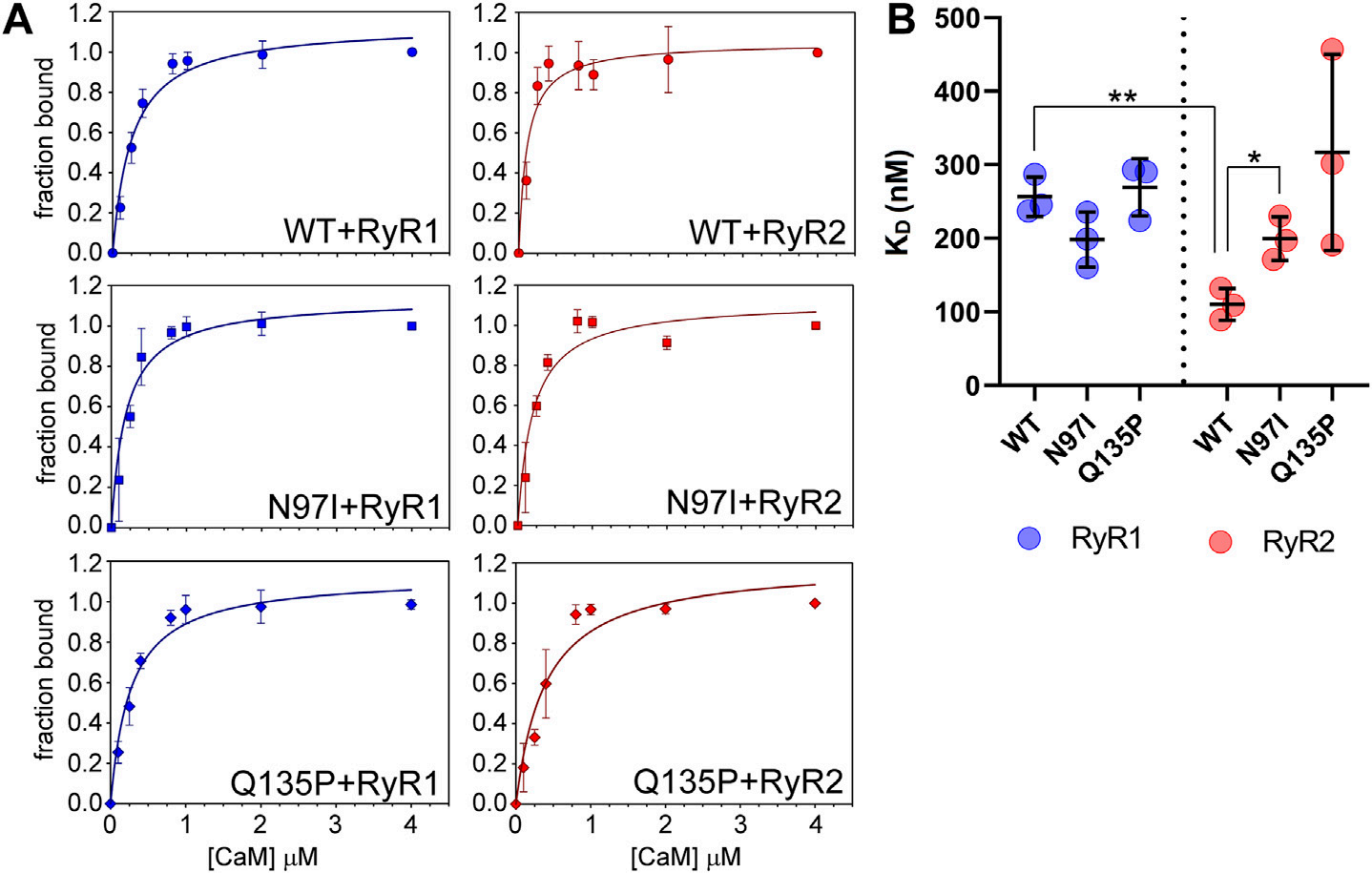
CaM affinity for RyR1 and RyR2 peptides assessed by fluorescence spectroscopy. **(A)** One micromolar RyR1 (left column) or RyR2 (right column) peptide was incubated with increasing amounts of WT (top row), N97I (middle row) and Q135P (bottom row) CaM in the presence of 100 µM Ca^2+^. Data are reported as a function of the peptide fraction bound to CaM (see Materials and Methods for details). Curves report the mean ± std of each point obtained in three technical replicas. Representative fitting to one-site saturation ligand binding curve is superposed to each titration set. **(B)** Scatter plot of replicates reporting the K_D_ values calculated from fitting procedures in each individual dataset using a one-site binding model. Stars represent *t*-test statistical significance: * *p*-value ≤ 0.05, ** *p*-value ≤ 0.01.

We took advantage of the lack of W residues in CaM and the presence of a single W residue in both RyR1 and RyR2 peptides to monitor the protein-peptide interaction by fluorescence spectroscopy, titrating a fixed amount of peptide with increasing amounts of CaM and evaluating the fraction of the peptide-bound protein in each condition (Figure 4A). This permitted an estimation of the apparent dissociation constant K_D_, reported in Table 1, which was used to quantify the affinity of each protein variant for the peptide. Interestingly, WT CaM showed a 2-fold higher affinity for RyR2 than RyR1 (K_D_^RyR2^ = 110 nM vs K_D_^RyR1^ = 256 nM, respectively; *p* < 0.01), while the two pathogenetic variants showed individually very similar affinities for each peptide (K_D_^RyR1^ = 198 nM vs K_D_^RyR2^ = 199 nM for N97I; K_D_^RyR1^ = 269 nM vs K_D_^RyR2^ = 317 nM for Q135P). The Q135P variant showed a more scattered behavior in the three replicates of RyR2 titrations compared to other variants (Figure 4B). It is worth noting that both pathogenetic variants resulted in significantly higher K_D_ values when titrated against RyR2 peptides compared to the WT (K_D_^WT^ = 110 nM vs K_D_^N97I^ = 199 nM, *p* = 0.013 and K_D_^Q137P^ = 317 nM, *p* = 0.056; Figure 4B and Table 1).

**TABLE 1:** Apparent affinities of CaM-RyR1/2 complexes assessed by fluorescence spectroscopy.

Variant	K_D_^RyR1^ (nM)	K_D_^RyR2^ (nM)
**WT**	256 ± 27	110 ± 22
**N97I**	198 ± 38	199 ± 30
**Q135P**	269 ± 39	317 ± 134

Overall, fluorescence spectroscopy measurements suggested that, besides a significantly decreased affinity for the RyR2 target compared to the WT, both N97I and Q135P pathogenetic variants apparently lose the capability to discriminate between RyR1 and RyR2 targets, a characteristic that was clearly observed for WT CaM.

**FIGURE 5:**
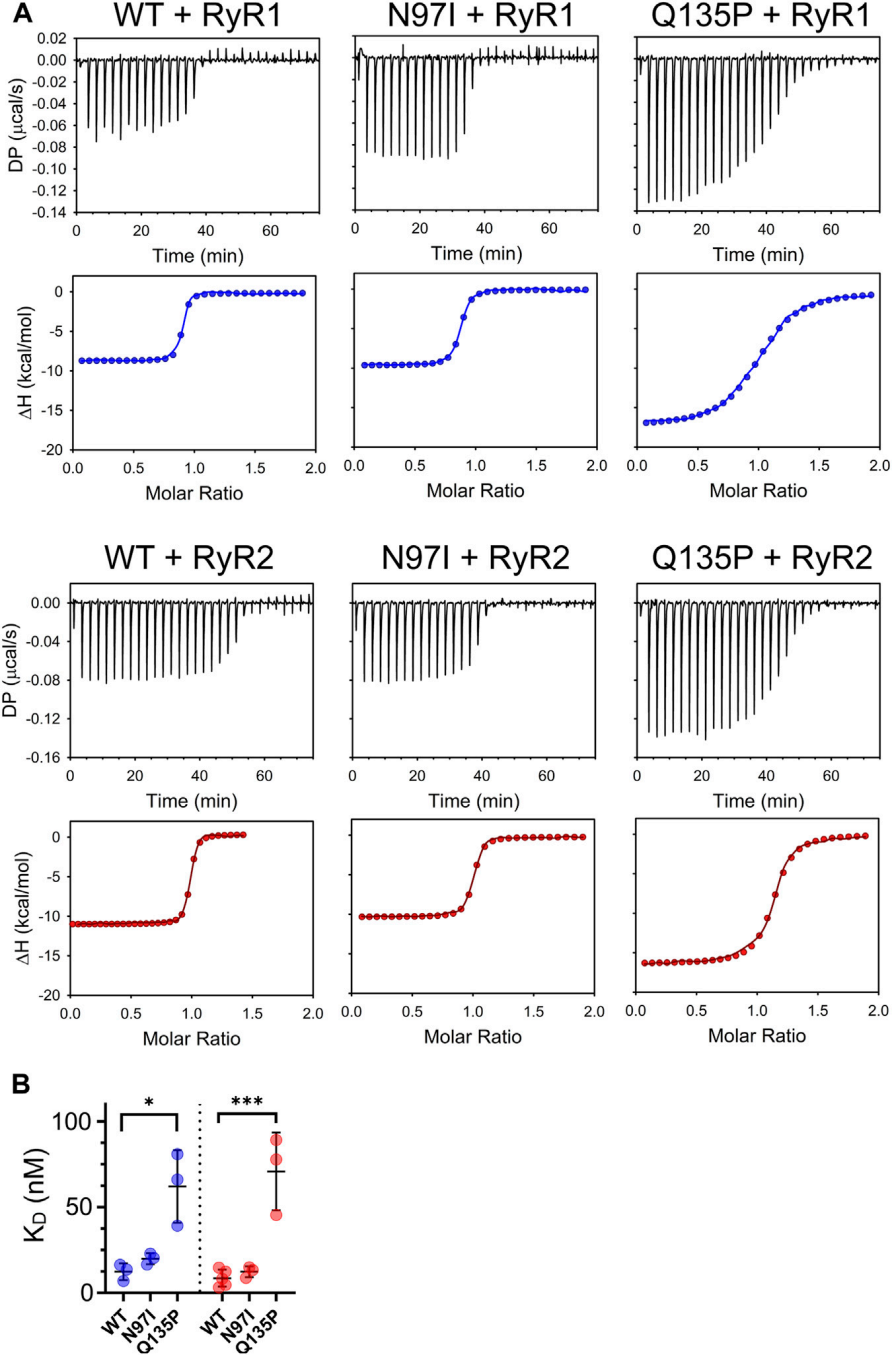
Thermodynamics of CaM-RyR1/2 peptide interaction assessed by isothermal titration calorimetry. **(A)** Examples of ITC titration curves obtained for each CaM variant upon interaction with RyR1 or RyR2 peptides. Measurements were performed at 25°C using 20 mM Tris pH 7.5, 150 mM KCl, 5 mM Ca^2+^ as working buffer and setting stirring at 750 rpm. Each titration consisted in thirty 1-µL injections of 125 µM RyR1 or RyR2 (into the titrant syringe) with 10 µM CaM variants. **(B)** Scatter plot of replicates summarizing the K_D_ values calculated from the fitting using a one-site binding model (see Materials and Methods). Stars represent the *p*-values: *p ≤ 0.05, ***p ≤ 0.001. Data for WT CaM titration with RyR2 are from (Dal Cortivo et al., 2022).

To probe the binding process by an essentially independent approach, we used isothermal titration calorimetry (ITC). In these experiments, either RyR1 or RyR2 peptides were titrated with a fixed amount of CaM variants (Figure 5). In line with our recent observations of other CaM variants titrated with RyR2 (Dal Cortivo et al., 2022), an exothermic binding process characterized by a 1:1 protein-peptide stoichiometry was detected in each case. Although the binding process was enthalpy-driven in each case, changes observed in the steepness of the transition (Figure 5A) indicated a mutation-specific affinity for each variant (Table 2). Interestingly, the binding of both RyR1 and RyR2 to Q135P CaM was entropically unfavored (10.14 kcal/mol vs 6.55 kcal/mol entropic contributions to ΔG, respectively). The affinity of WT CaM for RyR1 and RyR2 peptides was very similar (12.31 nM vs 8.62 nM, respectively, *p* = 0.34) while, interestingly, N97I showed higher affinity for RyR2 compared to RyR1 (K_D_^RyR1^ = 19.93 nM; K_D_^RyR2^ = 12.32 nM; *p* = 0.04). The Q135P pathogenetic variant showed again a more scattered interaction pattern with both peptides compared to other variants (Figure 5B) and the binding affinity was significantly reduced compared to the WT (K_D_^RyR1^ = 62.03 nM, *p* = 0.016; K_D_^RyR2^ = 83.45 nM, *p* = 0.008). ITC experiments thus essentially confirmed the results of fluorescence titrations, despite being less sensitive in detecting specific changes in affinity.

**TABLE 2:** Thermodynamics of CaM-RyR1/2 peptide association assessed by isothermal titration calorimetry.

Variant (n)	K_D_ (nM)	ΔH (kcal/mol)	ΔG (kcal/mol)	-TΔS (kcal/mol)	ΔΔG (kcal/mol)
RyR1					
** WT (3)**	12.31 ± 4.87	−9.94 ± 1.23	−10.78	−0.84	—
** N97I (3)**	19.93 ± 3.23	−9.58 ± 0.16	−10.50	−0.92	0.28
** Q135P (4)**	62.03 ± 21.13	−19.97 ± 4.37	−9.83	10.14	0.95
RyR2					
** WT** **^a^** **(5)**	8.62 ± 2.20	−11.28 ± 0.62	−11.01	0.27	—
** N97I (3)**	12.32 ± 3.18	−10.02 ± 0.13	−10.78	−0.76	0.23
** Q135P (3)**	83.45 ± 7.99	−16.20 ± 0.14	−9.65	6.55	1.36

n represents the number of independent titrations performed for each variant/peptide combination

^a^ These data are taken from (Dal Cortivo et al., 2022).

### 3.3 Mutation-specific effects on CaM-RyR1/2 interaction kinetics

**FIGURE 6:**
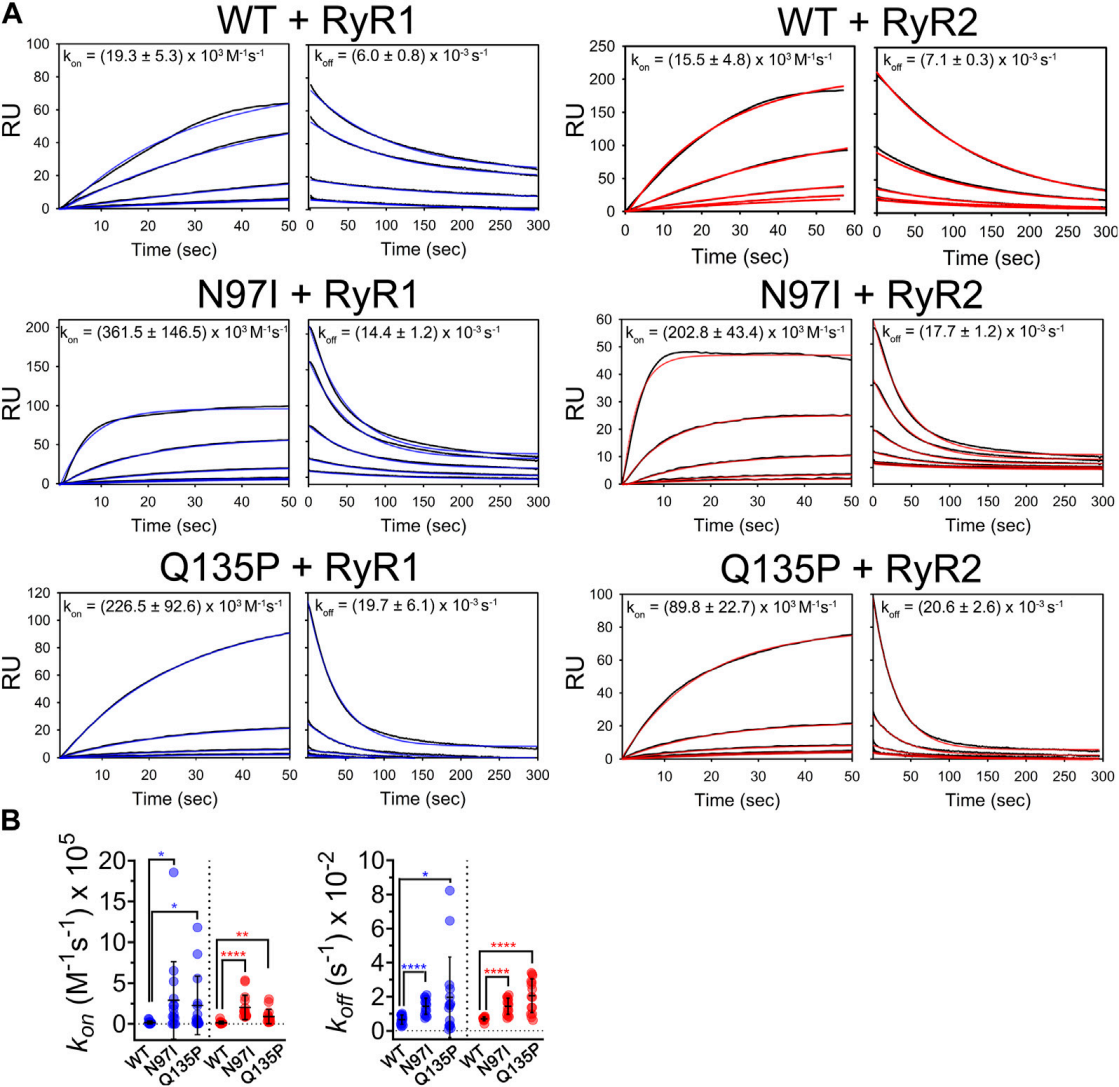
Kinetics of CaM-RyR1/2 peptide interaction investigated by surface plasmon resonance. **(A)** Sensorgrams collected by flowing different amounts of RyR1 and RyR2 (125 nM—2 µM) on immobilized His-CaM variants using 20 mM Tris pH 7.5, 150 mM KCl, 0.005% Tween 20, 5 mM Ca^2+^, 100 µM DTT as a running buffer. Association and dissociation phases were followed for 60 s and 300 s, respectively. Experimental curves (black solid lines) are shown together with theoretical curves (red or blue solid lines) according to a 1:1 Langmuir binding model; fitting for association and dissociation phases led to the rate constants (*k* ^on^ and *k* ^off^) reported in each panel (mean ± s.e.m. of 8-20 independent binding curves). **(B)** Scatter plot of replicates and statistical analysis comparing rate constants for WT and each pathogenic CaM variant. Stars represent *p*-values: *p ≤ 0.05, **p ≤ 0.01, ****p ≤ 0.0001.

Using a recently optimized procedure (Vallone et al., 2018; Dal Cortivo et al., 2019; Dal Cortivo et al., 2022), we immobilized CaM variants on the surface of a sensor chip through homogeneous histidine tag-mediated coupling. This allowed us to monitor the kinetics of the interaction with RyR1 and RyR2 peptides by surface plasmon resonance under Ca^2+^ saturating conditions. Increasing amounts of peptides were injected for 60 s on the chip where similar levels of CaM variants were previously immobilized to follow the association process, while the dissociation phase was monitored by flowing only running buffer for 300 s (Figure 6A). All sensorgrams could be fitted to a 1:1: Langmuir model compatible with a pseudo-first order association kinetics and a single exponential dissociation kinetics (Figure 6A, blue lines (RyR1) and red lines (RyR2)). Individual association (*k* ^*on*^) and dissociation (*k* ^*off*^) rate constants are reported in each panel of Figure 6A.

Kinetic analysis revealed an unexpected pattern. On one hand, when comparing the kinetics of CaM interaction with RyR1 and RyR2 peptides it was striking how the association and the dissociation process were unaffected by the presence or absence of point mutation, as the differences between *k* ^*on*^ and *k* ^*off*^ values for each RyR peptide were not statistically significant (Figure 6A, *p*-value ranging from 0.05 to 0.89 in all comparisons). However, it should be noted that upon interaction with RyR1 and RyR2, each pathogenic variant showed significantly different association and dissociation kinetics from WT (Figure 6B). Indeed, both N97I and Q135P CaM associated with RyR1, respectively, 19-fold faster (*p* = 0.027) and 12-fold faster (*p* = 0.029) than the WT. The same pattern was observed upon interaction with RyR2, where N97I CaM associated with the peptide 13-fold (*p* < 0.001) faster than the WT, while Q135P associated 6-fold faster (*p* = 0.0045) (Figure 6B). Differences in the dissociation rates of CaM-RyR complexes were even more significant. Both mutants dissociated faster than the WT from RyR1 and RyR2, the most significant differences being observed for the latter peptide (*k* ^*off*^_N97I_/*k* ^*off*^_WT_ = 2.5, *p* < 0.00001; *k* ^*off*^_Q135P_/*k* ^*off*^_WT_ = 2.9, *p* < 0.0001). In conclusion, kinetics of CaM-peptide recognition was found to be mutation-dependent, suggesting that selectivity for target is also based on kinetic discrimination.

### 3.4 Mutation-specific effects on the structural and dynamic properties of the CaM-RyR1/2 complexes monitored by MD simulations and free-energy calculations

To gain atomistic insights on the molecular properties detected for the arrhythmia-associated CaM variants, we ran extensive 1.2 µs MD simulations for each combination of CaM variants complexed with RyR peptides. The convergence and consistency of such simulations was assessed by analyzing the conformational space sampled by each replica using metrics derived by principal component analysis (PCA) of Cα motions, as described in Methods section. The overlap of the projection of the conformations sampled by the trajectories onto their first two principal components (Supplementary Figure S1), as well as of the density plots defined by the LDA classifier (Supplementary Figure S2) indicate that most conformations were accessible from different replicas, thus implying that all the replicas of each simulation were consistent. The same conclusion could be drawn from the comparison of the RMSIP (Supplementary Figure S3), which in all cases was found to be higher than 0.782, confirming that the replicas of MD simulations were reproducible and consistent, and therefore could be concatenated for further analyses.

**FIGURE 7:**
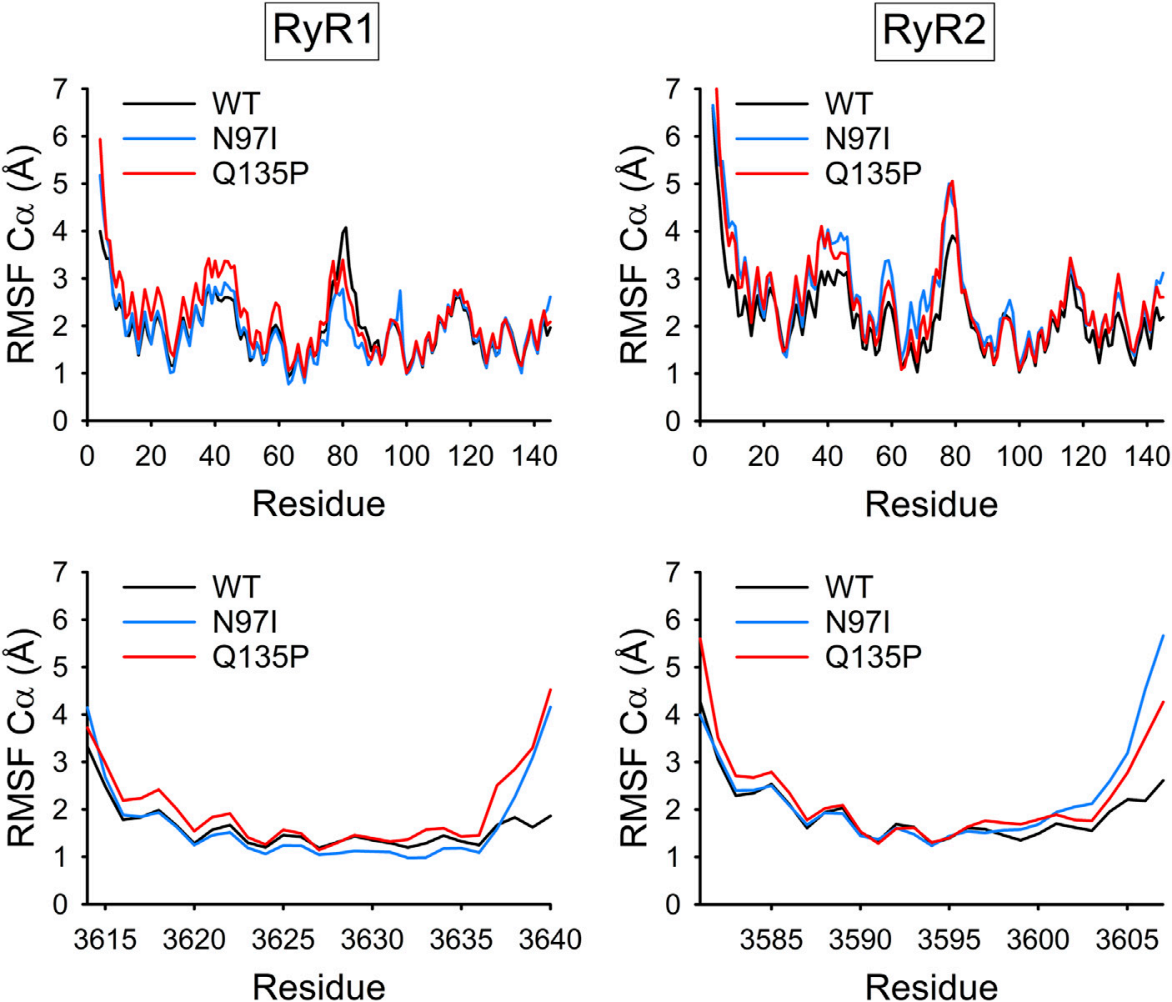
Backbone flexibility of CaM-RyR1/2 complexes. Cα-Root-Mean Square Fluctuation (RMSF) of CaM (top panels) and RyR1 or RyR2 peptides (bottom panels) calculated over 1.2 μs MD simulations of the respective complex (WT: black, N97I: blue, and Q135P: red).

The Cα-Root-Mean Square Fluctuation (RMSF) is a convenient index to evaluate the flexibility of the backbone of proteins or protein complexes, as it represents the root-mean square deviation of Cα atoms from the average structure mediated over the simulated timeframe. RMSF profiles suggest that CaM variants complexed to RyR1 peptide are very similar to the WT in terms of flexibility, with the largest differences observed in the N-terminal lobe (Figure 7, top panel) in particular for the Q135P substitution. The flexibility of the C-terminal domain was virtually unaffected by the mutations, which is surprising, as both mutations affect residues located at the C-terminal domain. A similar situation was observed with the CaM-RyR2 peptide complex, where a significantly higher flexibility was detected in the N-terminal lobe for both variants. Interestingly, the effects of the mutations on the flexibility of the backbone were significantly larger in this case, and, at odds with the CaM-RyR1 complex, the N97I variant exhibited the most prominent destabilization of the N-lobe. In contrast, the flexibility of both RyR1 and RyR2 peptides within each complex was essentially unaltered by the presence of pathogenetic variants, except for a lower flexibility shown by N97I CaM in complex with RyR1 (Figure 7, bottom panels).

The RMSF index can also be computed on Ca^2+^ ions to evaluate the tendency of cations to dislocate from the optimal bound geometry following mutations in the coordinating groups. Potential changes in cation binding affinity indeed reflect on the RMSF index, as a tight interaction of Ca^2+^ ions with its coordinating groups essentially leads to smaller fluctuations, while a loss of affinity would result in increased Ca^2+^ mobility around the optimal position (Marino et al., 2018). As to the CaM-RyR1 complex, both CaM variants exhibited no differences in Ca^2+^-coordination in EF3 and EF4 compared with WT CaM, even though substitutions were localized in these motifs (Supplementary Table S2). On the other hand, Ca^2+^-binding to EF1 and EF2 was significantly affected by the variants, suggesting an allosteric enhancement of the Ca^2+^-affinity in the case of the N97I mutant, at odds with the behavior displayed by the Q135P mutation (Supplementary Table S2). Overall, the Ca^2+^-RMSF profiles of CaM-RyR2 complexes suggested that Ca^2+^ may be significantly more loosely bound (∆RMSF ∼0.2 Å) with respect to CaM-RyR1 complexes in all simulated cases (Supplementary Table S2). While differences were almost negligible for the EF3-bound Ca^2+^ ion, fluctuations in EF2 and EF4 were significantly larger, suggesting a local, as well as a long-range negative effect of the mutations on Ca^2+^-affinity. Interestingly, in the presence of the Q135P substitution, also the Ca^2+^-ion in EF1 exhibited higher RMSF values compared to the WT, pointing again towards an allosteric rearrangement of the Ca^2+^-binding motif.

To dissect the effects of point mutations on the energetics of protein-peptide folding *versus* binding processes, we used a simplified thermodynamic cycle starting from the conformations sampled by MD simulations. This allowed us to estimate the apparent relative free energy of folding (ΔΔ $G_{\mathrm{app}}^{f}$ ) and binding (ΔΔ $G_{\mathrm{app}}^{b}$ ) for each variant/peptide (see Methods). The analysis suggested a stabilizing effect on the protein-peptide complex for the N97I substitution, both for RyR1 and RyR2 peptides (ΔΔ $G_{\mathrm{app}}^{f}$ = −24.10 kcal/mol and -5.58 kcal/mol, respectively, Supplementary Table S3). An opposite trend was detected for the Q135P substitution, which was predicted to significantly destabilize both complexes (ΔΔ $G_{\mathrm{app}}^{f}$ = 44.92 kcal/mol and 41.48 kcal/mol, respectively, Supplementary Table S3). Noteworthy, CaM variants carrying the N97I or Q135P substitutions displayed both a moderately increased apparent affinity for RyR1 (ΔΔ $G_{\mathrm{app}}^{b}$ = −0.47 kcal/mol and −0.15 kcal/mol, respectively), and moderately decreased apparent affinity for RyR2 (ΔΔ $G_{\mathrm{app}}^{b}$ = 0.13 kcal/mol and 0.53 kcal/mol), partly in line with fluorescence titration data. Taken together, modeling results suggest that CaM pathogenic variants perturb both the affinity for RyR peptides and the stability of the protein-peptide complex, but the specific effect appears to be mutation-dependent.

### 3.5 The topology of the protein-peptide structure network is altered by arrhythmia-associated mutations

The Protein Structure Network (PSN) defined by persistent non-bonded interactions between residues that occur during exhaustive MD simulations enables the study of the structural dynamics and the allosteric properties of Ca^2+^-sensor proteins (Marino and Dell'Orco, 2016; Marino and Dell'Orco, 2019). In addition, comparison of variant-specific PSNs provides information on the rewiring of those PSNs due to the presence of the mutation and thus the effects on the network topology.

**FIGURE 8:**
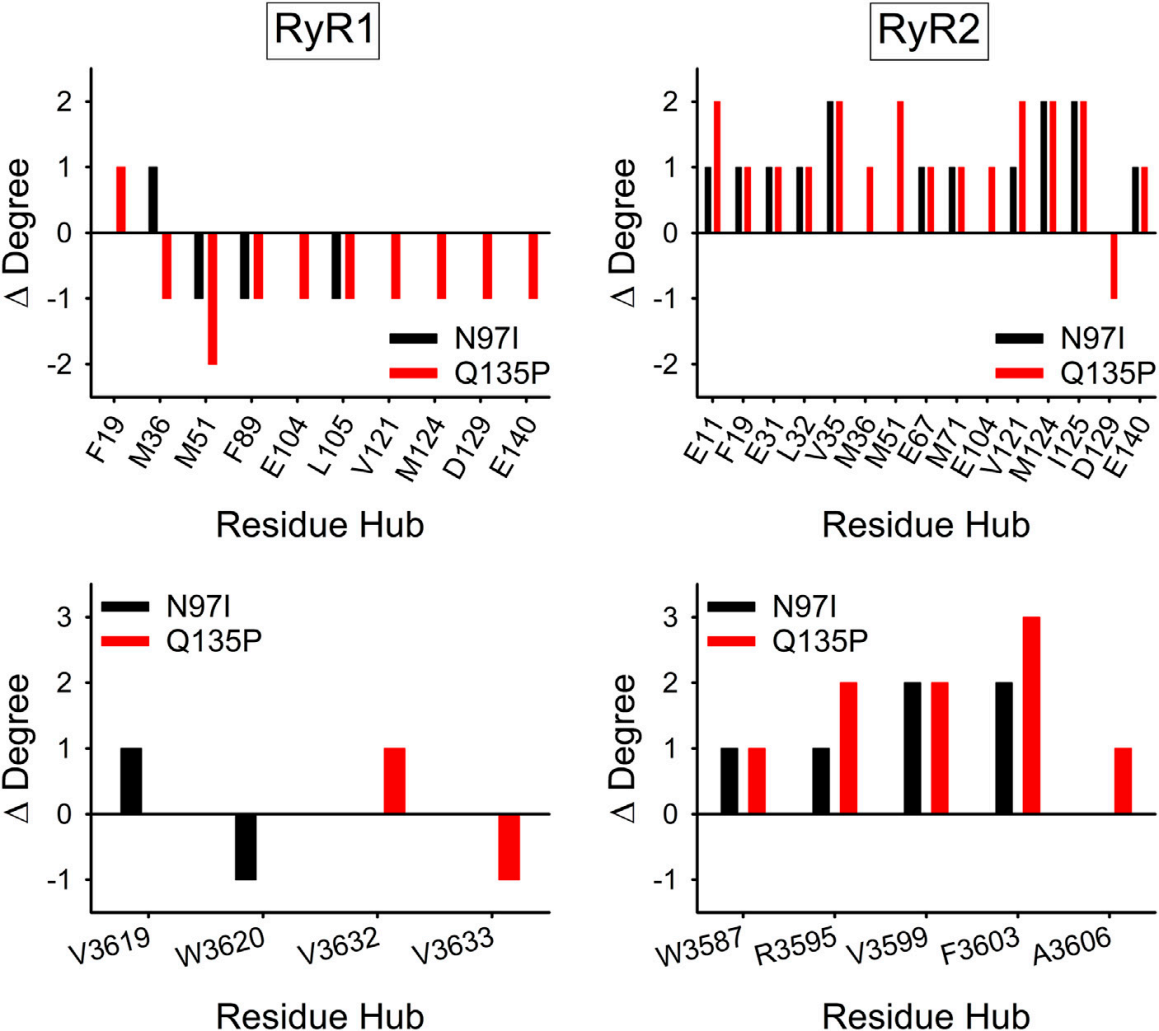
Effects of CaM variants on the connectivity of hub residues of CaM (top panels) and RyR1/2 peptides (bottom panels)**.** Hubs were defined as residues with degree ≥6 in the Protein Structure Network (PSN) of at least one variant. ∆Degree is calculated as the difference in hub degree between the variant (N97I: black, Q135P: red) and the WT.

Analysis of the variant-specific and target-specific PSN topology allows the identification of residues (hubs) involved in more than four persistent non-bonded interactions (hub degree) and thus responsible for preservation of protein structure, dynamics, and intra/intermolecular communications. Mutations affecting hub residues are more likely to destabilize the entire network than those affecting “peripheral” (i.e., less central) residues, involved in three or fewer interactions. Analysis of the change in connectivity of hub residues with degree ≥6 (Figure 8, top) suggested that in the presence of RyR1 the N97I variant slightly decreased the overall connectivity of these high-degree hubs (∆Degree = −2), with residues M51, F89, and L105 losing one interaction and residue M36 gaining one interaction compared with WT. On the other hand, the Q135P substitution significantly reduced the overall connectivity of the hubs (∆Degree = −9), with only F19 showing an additional interaction compared to the WT. As for the CaM-RyR2 complex, both N97I and Q135P variants surprisingly increased the overall degree of the hubs (∆Degree = 14 and 19, respectively), in clear contrast to the effects exerted on the CaM-RyR1 complex (Figure 8, top).

Considering only the hub residues belonging to RyR peptides, no change in overall connectivity in the CaM-RyR1 complex could be appreciated for both variants, with only two residues showing a ∆Degree = ± 1 in both cases (Figure 8, bottom). In contrast, in the CaM-RyR2 complex both variants exhibited a general increase in hub connectivity (∆Degree = 6 for N97I and 9 for Q135P), particularly in those located at the C-terminal of the peptide, namely R3595, V3599, F3603, A3606.

### 3.6 Unlike WT CaM, pathogenic variants are unable to discriminate between binding to RyR1 and RyR2

The allosteric properties of CaM should depend closely on its Ca^2+^-binding characteristics and are expected to reflect the existence of connecting routes of non-bonded interactions between its EF-hand Ca^2+^-binding motifs. We therefore searched for the existence of such persistent communication paths over the time frame of MD simulations. To this end, the intramolecular communication between the four EF-hands of CaM, represented by the 12^th^ residues (glutamate) of the Ca^2+^-binding loops was assessed by the Communication Robustness (CR) index, which takes into account the number and the length of the shortest paths between residues (see Methods).

**FIGURE 9:**
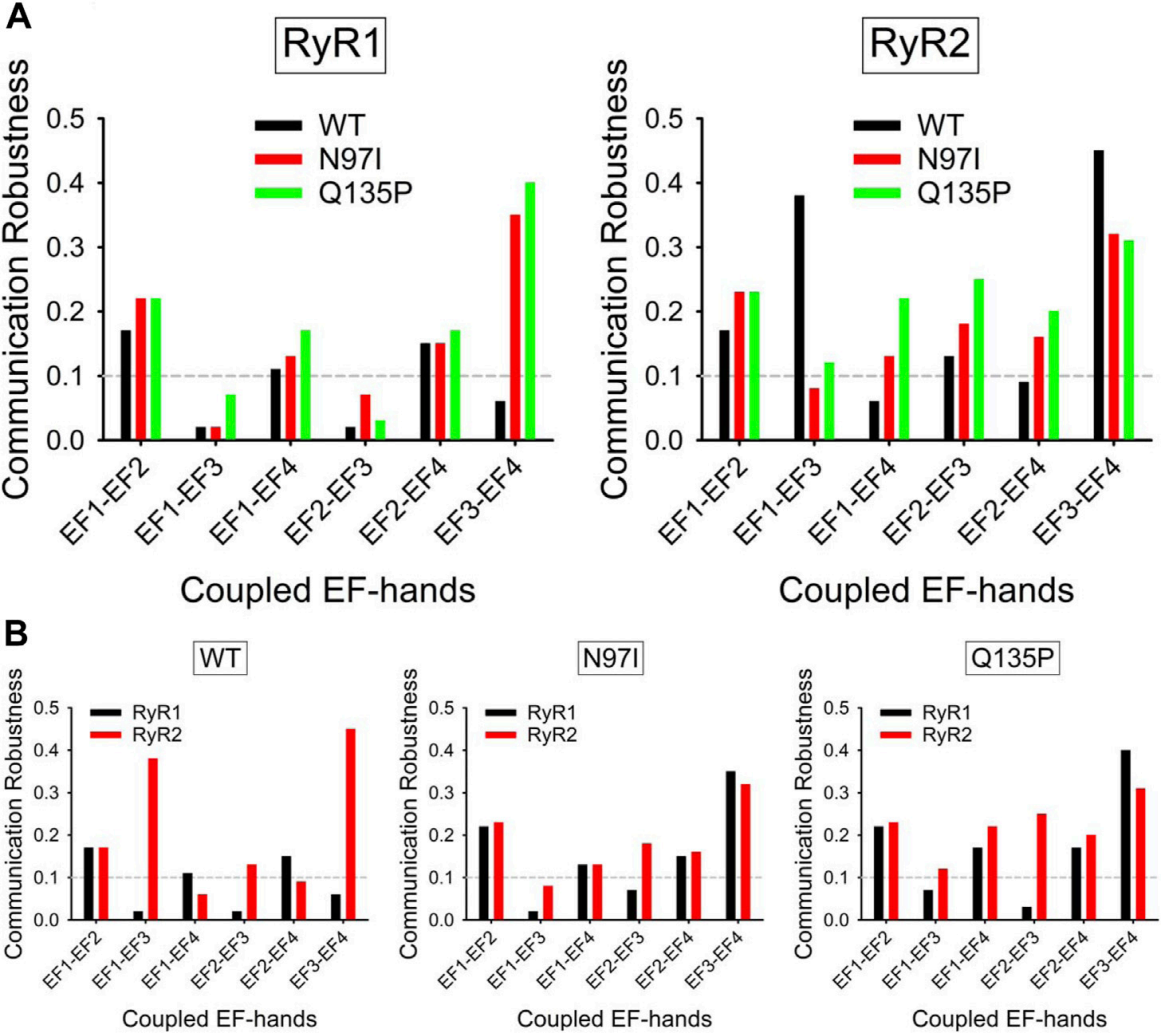
Robustness of intramolecular communication between EF-hands in CaM-RyR1/2 complexes. **(A)** Intramolecular communication among EF-hands in the CaM-RyR1 (left) and CaM-RyR2 (right) complexes. Communication robustness between EF-hands (EF1 to EF4, represented by their bidentate Glu residues) in CaM WT (black), N97I (red), and Q135P (green) variants in complex with RyR peptides. **(B)** Effects of specific RyR peptides on the intramolecular communication among EF-hands in CaM-RyR complexes. Communication robustness between EF-hands (EF1 to EF4, represented by their bidentate Glu residues) in CaM variants in complex with RyR1 (black) and RyR2 (red) peptides.

In the CaM-RyR1 peptide complexes, all variants showed a robust communication between EF1-EF2, EF1-EF4, and EF2-EF4 (Figure 9A), and no robust communication between EF1-EF3 and EF2-EF3 (CR threshold for significant robustness was set at 0.1). Interestingly, the only significant difference between WT and pathogenic CaM variants was observed in the EF3-EF4 communication, which was absent in the WT (CR < 0.1) but very robust in the case of both variants (CR > 0.3). When the same analysis was performed for CaM-RyR2 peptide complex, a pattern of higher robustness in communication between EF-hand pairs was found for both pathogenic variants, which showed higher CR indexes in all cases except for EF1-EF3 communication. While qualitatively in line with the increased connectivity observed for CaM-RyR2 hubs in the presence of pathogenic mutations (Figure 9B), this analysis revealed another important feature in terms of specificity in target recognition. Indeed, WT CaM was the only variant able to discriminate RyR1 and RyR2 targets through the appearance of allosteric communication between EF1-EF3 and EF3-EF4 (Figure 9B), as the communication between this specific EF-hand pairs was very significant (CR ∼ 0.4) only when in complex with RyR2, whereas it was essentially lost when binding to RyR1 (CR < 0.2, Figure 9B). In contrast, in the presence of either RyR peptide, both N97I and Q135P CaM variants showed non-robust communication between EF1-EF3, and a highly robust (CR > 0.3) communication between EF3-EF4, thus losing any ability to discriminate between targets.

## 4 Discussion

The redundancy of genes encoding CaM in the human genome, its high conservation among the reigns, and its promiscuity in terms of molecular partners all suggest that missense mutations in the genes encoding CaM be deleterious and probably incompatible with life. However, the finding that 17 CaM-missense mutations are associated with lethal arrhythmia and lead to no other apparent phenotype suggests the existence of a specific effect of disease-associated CaM variants on a target that is intimately connected with pathology. In other words, CaM variants associated with LQTS and CPVT must recognize RyR channels in skeletal (RyR1) and cardiac (RyR2) muscle cells differently from WT CaM. Several lines of evidence suggest that the CaM mutants analyzed in this study (N97I and Q135P) cause arrhythmia through dysregulation of RyR2 function. Indeed, the N97I substitution results in an altered interaction with the IQ domain of the cardiac voltage-gated calcium channel (Wang et al., 2018), but on the other hand, another substitution at the same locus, i.e. N97S, was found to affect RyR2-mediated calcium release, specifically increasing it compared to WT CaM (Sondergaard et al., 2015). A direct perturbation of the interaction with RyR2 is therefore expected for the N97I variant. Similarly, the Q135P substitution was found to affect binding to the IQ domain of Cav1.2 (Wang et al., 2020), but also the activation threshold of RyR2, thus likely promoting spontaneous Ca^2+^ release in cardiomyocytes during diastoles (Sondergaard et al., 2019). The involvement of these CaM variants in RyR2 dysregulation thus appears substantial.

Our structural analysis clearly showed that the N97I and Q135P substitutions lead to small but appreciable alterations of protein secondary and tertiary structure. Such effects are substantially in line with previous observations by NMR and X-ray crystallography that individual mutations perturb the conformation of CaM and its interaction with the target peptide, in a mutation-specific manner (Wang et al., 2018; Dal Cortivo et al., 2019). A common fingerprint linking arrhythmia-associated variants N53I in EF2 (Holt et al., 2020) with D95V/H in EF3 and D131V/H/E in EF4 (Dal Cortivo et al., 2022) seems to be the fact that the single mutation can perturb to various extent protein tertiary structure, but all variants alter the protein intramolecular dynamics, affecting and destabilizing the N-terminal domain, which has been suggested to permit the functional recognition of RyR2 target *via* allosteric interactions (Westerlund and Delemotte, 2018; Dal Cortivo et al., 2022). Clearly, understanding the ability of CaM variants to discriminate between specific targets requires a complementary approach to the structural analysis, and requires full characterization of the binding process. Considering the very high sequence similarity between RyR1 and RyR2 CaM-binding domains investigated in this study, we performed an exhaustive characterization using several techniques.

The W residue in the RyR2 (and RyR1) peptide mimicking the channel region interacting with CaM has been shown to be essential for the molecular recognition both at low and high Ca^2+^ levels (Brohus et al., 2019). Titrations experiments based on intrinsic W fluorescence showed that WT CaM binds RyR2 with double affinity compared to RyR1, which is quite surprising considering the minor difference in the peptide sequences, and indicates a high target selectivity. On the other hand, sensitivity of ITC experiments did not allow to distinguish the affinity of WT CaM for RyR1 and RyR2 peptides. Care should be taken when interpreting K_D_ values obtained by ITC. It has previously been shown for calcium sensor proteins, which undergo significant conformational changes upon Ca^2+^ and target binding, that the apparent K_D_ value obtained by ITC may not represent the true dissociation constant. In fact, in ITC titrations, an enthalpic factor related to conformational changes is inevitably added to the enthalpy change of the binding process (Dell'Orco et al., 2010; Dell'Orco et al., 2012) and cannot be distinguished from the pure ΔH of binding. Since RyR peptides fold upon binding, it is thus not surprising that ITC does not fully discriminate between different RyRs. Our ITC results are also qualitatively in line with previous ones obtained by (Lau et al., 2014), who measured a 46 nM affinity for RyR1 and 47 nM affinity for RyR2. The slight differences can be attributed to different experimental conditions, including shorter RyR1 and RyR2 peptides lacking the -LYNL sequence at the C-terminal and different buffer used in (Lau et al., 2014). ITC patterns in our study and the emerging thermodynamics are also similar to those obtained for N53I (Holt et al., 2020) and F141L (Sondergaard et al., 2017) arrhythmogenic CaM variants binding to RyR2 peptides and with the results obtained very recently by us with the D95V/H, D129V, and D131H/E arrhythmogenic variants (Dal Cortivo et al., 2022).

While useful to reveal intrinsic perturbations in the CaM-peptide binding thermodynamics associated with pathogenic mutations, ITC experiments are evidently insufficient to fully describe the recognition between CaM and very similar targets, and point to the necessity of going beyond characterization of the equilibrium to assess the determinants of target discrimination. Kinetic characterization of the interaction between CaM variants and RyR1 and RyR2 peptides showed that both N97I and Q135P pathogenic variants associated with RyR1 and RyR2 peptides significantly faster than the WT, however their dissociation from the RyR2 peptide was more than two-fold faster than WT CaM, suggesting the involvement of kinetic discrimination in target selectivity.

A deeper insight into the recognition process emerged from MD simulations and analysis of RMSF profiles, which suggested that Ca^2+^-binding affinities in specific EF-hands can be modulated by the pathogenic variants even when mutations are located far away from the binding sites. This would be possible if allosteric mechanisms mediated the structural communication between EF-hands, not only within the same domain, but also between different domains. It is relevant that both N97I and Q135P substitutions, located respectively in EF3 and EF4, reflect in a perturbation of the flexibility of EF2 and EF1 Ca^2+^ binding loops in the presence of RyR1 and RyR2 peptide, and specifically, increase the flexibility in this latter case. This finding is especially interesting considering previous results that suggest that the functional recognition between CaM and its RyR target involves allosteric interactions initiated by the N-terminal lobe of CaM (Dal Cortivo et al., 2022), in spite of its lower affinity for Ca^2+^. It has been postulated that long-range electrostatic interactions of amino acids at the N-terminal domain are responsible for initiating CaM binding to the target, while short-range hydrophobic interactions in the C-terminal lobe may account for selectivity (Westerlund and Delemotte, 2018). Our simulations indeed support this view.

Interesting topological properties, with deep implications for target selectivity emerged from PSN analysis. Our data indeed indicate as a peculiarity of the pathogenic variants the significantly increased connectivity in the PSN formed by the complex of CaM with RyR2, which is the target channel in pathologic conditions. This increased connectivity evidently extends beyond the intramolecular communication and reaches protein-target intermolecular contacts, thus inducing a complete rewiring of the network in the co-presence of pathogenic mutations and cell-specific target. Finally, topological analysis of the structural dynamics that emerged from MD simulations suggests that the ability of WT CaM to discriminate highly similar RyR1 and RyR2 targets is based on the robust allosteric communication between EF1-EF3 and EF3-EF4 pairs in CaM-RyR2 recognition, the former of which is lost in both N97I and Q135P variants.

In conclusion, we presented an in-depth characterization of the recognition between a common CaM-binding region in RyR1 and RyR2 and two arrhythmia-associated CaM variants in their Ca^2+^-bound states. In a broader context, our analysis suggests that a complex combination of factors may influence the discrimination ability of CaM towards its many targets, which includes kinetic discrimination and specific allosteric communication between its Ca^2+^-binding EF-hand motifs.

## Data Availability

The original contributions presented in the study are included in the article/Supplementary Material, further inquiries can be directed to the corresponding author.
