# Supplementary material for: Calmodulin variants associated with congenital arrhythmia impair selectivity for ryanodine receptors
Source: Front Mol Biosci. 2023 Jan 5;9:1100992. doi: 10.3389/fmolb.2022.1100992 (PMC9849693; doi:10.3389/fmolb.2022.1100992)
Supplement: Supplementary file 1 [file DataSheet1.pdf]

Supplementary Material

Calmodulin variants associated with congenital arrhythmia impair selectivity for ryanodine receptors

Giuditta Dal Cortivo, Valerio Marino, Silvia Bianconi, Daniele Dell'Orco

1 Supplementary Tables

Table S1: Spectroscopic features of CaM-RyR peptides complexes assessed by far-UV CD spectroscopy

		WT			N97I			Q135P		
		**Alone**	**+ RyR1**	**+ RyR2**	**Alone**	**+ RyR1**	**+ RyR2**	**Alone**	**+ RyR1**	**+ RyR2**
**$\theta_{222}/\theta_{208}$[a]**	Apo	0.91	0.88	0.92	0.93	0.89	0.87	0.93	0.90	0.90
	Ca^{2+}	0.99	0.98	1.00	0.97	0.99	0.96	0.90	1.02	0.99
**$\Delta\theta_{222}/\theta_{222}$ (%)[b]**		18	28	25.5	8.6	16.7	20	-11.6	22.3	16.6

[a] Ratio between ellipticity at 222 and 208 nm

[b] $\Delta\theta_{222}/\theta_{222}$ is calculated as $(\theta_{222}^{Ca2+} - \theta_{222}^{apo})/\ \theta_{222}^{apo}$

Table S2: Root-Mean Square Fluctuations of Ca^{2+} ions (Å) bound to individual EF-hands of CaM variants in CaM-RyR peptides complexes

	**RyR1**			**RyR2**		
**Ca^{2+}-ion**	**WT**	**N97I**	**Q135P**	**WT**	**N97I**	**Q135P**
**EF1**	1.594	1.469 ↓	1.985 ↑	2.064	2.015	2.224 ↑
**EF2**	1.379	1.186 ↓	1.56 ↑	1.603	2.172 ↑	1.786 ↑
**EF3**	1.48	1.473	1.492	1.591	1.563	1.501
**EF4**	1.555	1.53	1.571	1.645	1.941 ↑	2.27↑
**Average**	1.502	1.415 ↓	1.652 ↑	1.726	1.923 ↑	1.945 ↑

Table S3: Effects of arrhythmia-associated variants on the relative apparent stability ($\Delta\Delta G^{f}_{app}$) and affinity ($\Delta\Delta G^{b}_{app}$) of the CaM-RyR peptides complexes Data refer to the average ± standard error of the values calculated on the final structure of each of the four replicas.

	**RyR1**		**RyR2**	
	**$\Delta\Delta G^{f}_{app}$ (kcal/mol)**	**$\Delta\Delta G^{b}_{app}$ (kcal/mol)**	**$\Delta\Delta G^{f}_{app}$ (kcal/mol)**	**$\Delta\Delta G^{b}_{app}$ (kcal/mol)**
**N97I**	-24.10 ± 17.12	-0.47 ± 0.24	-5.58 ± 1.16	0.13 ± 0.08
**Q135P**	44.92 ± 2.45	-0.15 ± 0.14	41.48 ± 11.52	0.53 ± 0.19

2 Supplementary Figures

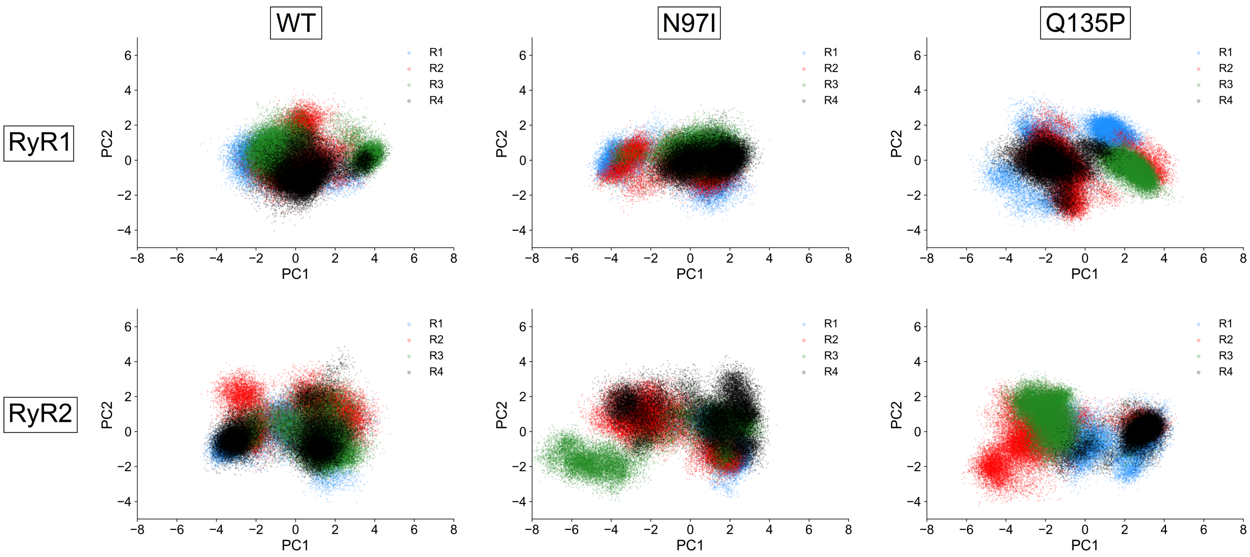

Figure S1. Consistency the conformational space sampled by independent MD replicas of CaM variants - 1. The frames of the four 300 ns replicas (R1: blue, R2: red, R3: green, and R4: black) were projected onto the first two principal components (PC1 and PC2) representing the largest collective motion of the protein. The principal components were calculated on the covariance matrix of Cα on the concatenated 1.2 µs trajectories of CaM WT, N97I, and Q135P in the presence of either RyR1 or RyR2 peptides.

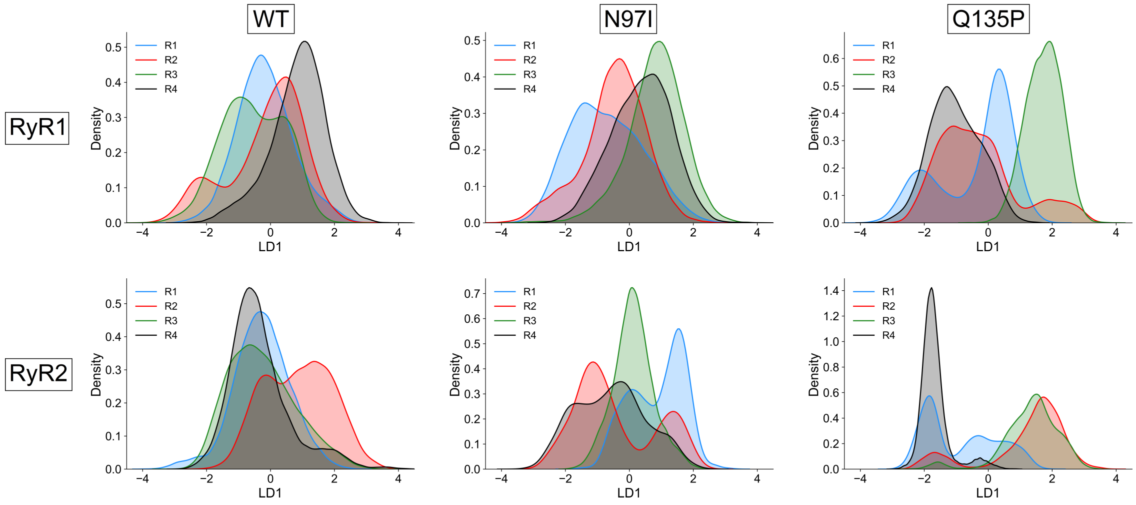

Figure S2. Consistency the conformational space sampled by independent MD replicas of CaM variants - 2. Linear Discriminant Analysis of the frames of the four 300 ns replicas (R1: blue, R2: red, R3: green, and R4: black) projected onto the first two principal components calculated on the concatenated 1.2 µs trajectories of CaM WT, N97I, and Q135P in the presence of either RyR1 or RyR2 peptides.

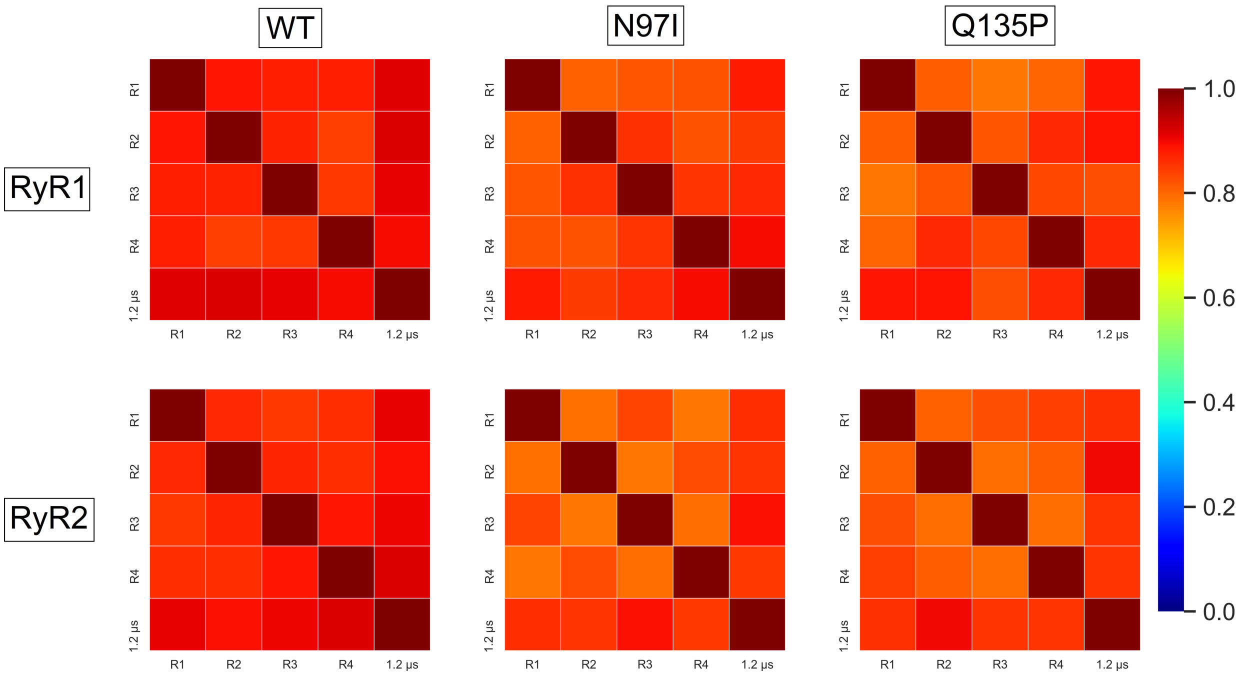

Figure S3. Consistency the conformational space sampled by independent MD replicas of CaM variants - 3. Root-Mean Square Inner Product (RMSIP) of the first 20 principal components calculated on the 4 single replicas and on the concatenated 1.2 µs trajectories of CaM WT, N97I, and Q135P in the presence of either RyR1 or RyR2 peptides. RMSIP values are displayed in a rainbow color scale from 0 to 1.
